# Cost analysis of the management of end-stage renal disease patients in Abuja, Nigeria

**DOI:** 10.1186/s12962-023-00502-3

**Published:** 2023-12-08

**Authors:** Yakubu Adole Agada-Amade, Daniel Chukwuemeka Ogbuabor, Ejemai Eboreime, Obinna Emmanuel Onwujekwe

**Affiliations:** 1 Department of Health Administration and Management, University of Nigeria, Enugu Campus, Nigeria Enugu, Enugu State Nigeria; 2 National Health Insurance Authority, Abuja, Nigeria; 3 Department of Health Systems and Policy, Sustainable Impact Resource Agency, Enugu, Nigeria; 4 Department of Psychiatry, Faculty of Medicine, Dalhousie University, Halifax, NS Canada; 5 Health Policy Research Group, Department of Pharmacology and Therapeutics, College of Medicine, University of Nigeria Enugu Campus, Enugu, Nigeria

**Keywords:** End-stage renal Disease, Haemodialysis, Costs, Economic evaluation

## Abstract

**Background:**

Although the treatment for end-stage renal disease (ESRD) under Nigeria’s National Health Insurance Authority is haemodialysis (HD), the cost of managing ESRD is understudied in Nigeria. Therefore, this study estimated the provider and patient direct costs of haemodialysis and managing ESRD in Abuja, Nigeria.

**Method:**

The study was a cross-sectional survey from both healthcare provider and consumer perspectives. We collected data from public and private tertiary hospitals (n = 6) and ESRD patients (n = 230) receiving haemodialysis in the selected hospitals. We estimated the direct providers’ costs using fixed and variable costs. Patients’ direct costs included drugs, laboratory services, transportation, feeding, and comorbidities. Additionally, data on the sociodemographic and clinical characteristics of patients were collected. The costs were summarized in descriptive statistics using means and percentages. A generalized linear model (gamma with log link) was used to predict the patient characteristics associated with patients’ cost of haemodialysis.

**Results:**

The mean direct cost of haemodialysis was $152.20 per session (providers: $123.69; and patients: $28.51) and $23,742.96 annually (providers: $19,295.64; and patients: $4,447.32). Additionally, patients spent an average of $2,968.23 managing comorbidities. The drivers of providers’ haemodialysis costs were personnel and supplies. Residing in other towns (HD:β = 0.55, ρ = 0.001; ESRD:β = 0.59, ρ = 0.004), lacking health insurance (HD:β = 0.24, ρ = 0.038), attending private health facility (HD:β = 0.46, ρ < 0.001; ESRD: β = 0.75, ρ < 0.001), and greater than six haemodialysis sessions per month (HD:β = 0.79, ρ < 0.001; ESRD: β = 0.99, ρ < 0.001) significantly increased the patient’s out-of-pocket spending on haemodialysis and ESRD.

**Conclusion:**

The costs of haemodialysis and managing ESRD patients are high. Providing public subsidies for dialysis and expanding social health insurance coverage for ESRD patients might reduce the costs.

## Background

Chronic kidney disease (CKD) represents a substantial burden in low and middle-income countries (LMICs), which are less equipped to deal with its consequences [1]. End-stage renal disease (ESRD) is a significant consequence of CKD, requiring long-term haemodialysis of 2–3 sessions per week [2]. Chronic kidney disease entails kidney damage with glomerular filtration rate (GFR) of < 60 mL/1.73 m for 3 months or more, irrespective of the cause. ESRD is defined as loss of kidney function of GFR < 15 mL/ 1.73 m [3]. Without kidney replacement therapy, such as haemodialysis, ESRD remains uniformly fatal [4]. End-stage renal disease is a regional public health epidemic in sub-Saharan Africa with an unacceptably high cost of management [5]. In Africa, the annual cost varies from $7,370 to $42,800 per patient [6], far more than most African countries’ gross national product (GDP) per capita. The cost of haemodialysis varies geographically and between the public and private providers of renal care services in Nigeria. The cost per session ranges between $62 and $250 or an average of $744 to $3,000 monthly, assuming three times weekly sessions, while the monthly minimum wage in Nigeria is below $100 [7]. Uncontrolled comorbidities with diabetes, hypertension, and other diseases increase the cost of haemodialysis among ESRD patients [8]. Without public subsidy, most ESRD patients cannot afford dialysis and the medications associated with it [2, 5]. Accordingly, the demand for haemodialysis might decrease when its costs increase, resulting in poor retention in care.

Including new interventions and technologies in benefit packages of universal coverage schemes based entirely on clinical evidence without considering cost issues may not be optimal [9]. Despite an increasing number of health facilities offering dialysis in Nigeria, the high cost of services still poses a considerable barrier to accessing care [10, 11]. Unsurprisingly, out-of-pocket payment remains the predominant source of healthcare financing in the Country [12]. To reduce the financial burden on consumers, the National Health Insurance Authority (NHIA) introduced the coverage of renal dialysis to the benefit package of its programme, the Formal Sector Social Health Insurance Programme (FSSHIP), in 2012. The intervention is, however, limited to partial coverage (50%) for six sessions, leaving the burden of having to pay 50% of the cost of dialysis to the patient. After the first six sessions, the patient pays entirely out-of-pocket. Evidence shows that most Nigerians requiring haemodialysis could not sustain three sessions per week beyond a few weeks due to insufficient funds [2]. Therefore, costing dialysis and other treatment modalities is required to understand the budget impact of coverage by government or third-party administrators.

Most studies indicate that the cost of haemodialysis among ESRD patients is high from both the provider perspective [3, 6, 13–16], patient perspectives [2, 16–18], and uncertain [19, 20]. Variations in cost are due to variable patient management protocols, currency volatility, timing of studies, and methodology when assessing costs [6]. The main health systems’ cost drivers are supplies and human resources [14, 19]. Direct medical costs constitute most patient costs [13, 17]. Financial barriers limit most ESRD patients from sustaining long-term haemodialysis [21]. Increasing the number of haemodialysis sessions raises the cost from the patient’s perspective [17]. In Nigeria, discontinuing haemodialysis is associated with the inability to pay for haemodialysis more than once per week [2]. Some studies have proposed twice-weekly dialysis to lower the total cost of haemodialysis since twice-weekly dialysis has non-inferior survival rates compared with thrice-weekly therapy [15, 22, 23]. Other drivers of patient costs include comorbidities with additional chronic diseases [3, 15, 17, 18, 24, 25], use of publicly owned university hospitals [26], attending private health facilities [27], age [17, 25], residence environment [18, 24, 26], income and wealth index [13, 17, 19]. Few studies indicate that national health insurance improved accessibility to health service utilization for household members with chronic kidney disease by reducing direct medical costs [28, 29].

This article is part of a broader economic evaluation study that examined the cost and benefits of dialysis and the treatment modalities for ESRD available on the benefit package of the NHIA. Our basis for measuring the costs of treating ESRD with haemodialysis is that resources are scarce, and there are competing alternatives to using available healthcare resource [30]. The study considered the demand and supply sides of managing ESRD by using direct cost data from both the patient and provider perspectives. The aim was to determine the cost of haemodialysis to the patient and providers alike. Additionally, the study assessed the factors associated with patient costs. Policymakers can use this information to plan for dialysis services, set provider payment rates for dialysis under various publicly sponsored health insurance schemes, and guide resource allocation decisions.

## Methods

### Study area and population

This study took place in Abuja, Nigeria’s federal capital territory (FCT). The city, which has a population of 3.84 million, has 15 hospitals offering renal dialysis including nine private, five public, and one public-private partnership hospital [31]. Abuja was chosen for this study because it has the highest FSSHIP enrolment and about 19% of the 80 actively functioning dialysis centres in Nigeria [32]. Though second to Lagos State in the number of dialysis centres, Abuja’s dialysis centres serve Nigeria’s entire North-Central zone, unlike Lagos State, which primarily serves its residents. We purposively chose six (6) of the 15 hospitals (three public, two private and one public-private partnership) to ensure maximum variation based on type, ownership and geographical locations. Nigeria’s National Health Insurance Authority accredited all the selected hospitals. About 63% of Nigerians are multidimensionally poor and cannot afford regular hospital [33].

The study population was chronic kidney disease patients with ESRD accessing dialysis care in Abuja and healthcare facilities supplying such care during the study recruitment period. We included ESRD patients who do not live but accessed care in Abuja. The study excluded ESRD patients residing in Abuja but undergoing renal dialysis outside the territory since our study was hospital-based study.

### Study design

We conducted a hospital-based study using a cross-sectional survey design to collect cost data from provider and patient (consumer) perspectives.

### Sample size and sampling strategy

The calculated sample size for this study was 165 using the sample size determination formula for infinite proportion, given a prevalence of chronic kidney diseases of 10.76 in Abuja, 95% confidence limit, allowable error of 0.05 and 10% non-response rate. The sampling strategy was a multistage sampling technique using a sampling frame of 15 facilities that offer renal services in Abuja. We used stratified random sampling to select public/private/public-private partnerships (PPP) and facilities in suburban/rural areas and urban areas. Simple random sampling was then adopted to select four healthcare facilities in urban/municipal areas and two in satellite towns. The stratification ensured that the recruitment of facilities reflected geographical location and ownership. We selected eligible patients by simple random sampling.

### Data collection procedure

Data was collected using three different interviewer-administered questionnaires: scoring cost of renal dialysis, healthcare providers cost survey, and patient direct cost and equity questionnaires. The scoring cost of renal dialysis, adopted from a previous study [34], was used to ascertain the most suitable cost items to include in the provider questionnaire. The researcher administered the tool through email, telephone, or face-to-face interviews with nephrology practitioners (n = 25), including nephrologists, nurse nephrologists, technicians, laboratory scientists, health economists and health administrators. We assessed each cost item on four domains: feasibility, importance, contextual relevance, and frequency of use. Each domain was scored on a 5-point Likert scale (poor = 1, somewhat poor = 2, somewhat good = 3, good = 4, and very good = 5). The composite score for each item ranged from 0 to 20. We retained all the items with composite scores greater than or equal to 12. Furthermore, using the scoring, we determined the scale content validity index of the providers’ cost survey tool to be 0.93.

The healthcare provider cost survey was adapted from Mushi et al [6] and further modified with findings of the scoring cost of the renal dialysis tool. The provider cost tool collected data on variable and fixed cost items. An estate valuer estimated the cost of rent or building. The study estimated the dialysis unit space’s cost by obtaining the unit’s surface area against the entire hospital building. The salaries of the nephrologists, medical doctors/registrars, nurse-nephrologists, nurses, nutritionists, laboratory scientists, health attendants and technicians were included in the variable cost based on the time spent on renal dialysis. The patient direct cost and equity questionnaires collected patient data on cost, equity, and affordability. The patient’s questionnaire had three sections. The first section collected sociodemographic data, including age, gender, marital status, residence, employment, income, and education. The second part collected wealth index data using the Nigeria equity tool developed by the Metrics for Management [35]. The third part collected data on facility type, number of haemodialysis sessions, type of arteriovenous access, cost of haemodialysis, and cost of comorbidities. Data were collected directly by the lead researcher and five research assistants. The research assistants received training on the tools and research ethics for two days, including pre-testing the tools. Data were archived in a secure computer anonymously.

### Data analysis

The study analyzed the data with SPSS version 20. In converting Nigerian Naira () to US dollars ($), the study used an exchange rate of 305.8 to $1 (2018). We annualized fixed costs to their annual equivalents and implicit rental values by discounting costs at 3%. The cost function for this study described the total cost y, the sum of the total fixed costs ‘a’, and the total variable costs ‘b’, in the equation y = a + bx. The factor ‘x’ is the number of units produced. In analyzing the providers ' costs, we assumed an average of three sessions per week for 52 weeks, which is 156. Therefore, we multiplied the unit cost by 156 to estimate the annual costs. We summarized the providers’ costs using descriptive statistics, including means, standard deviation, and percentages.

The socio-demographic characteristics of respondents were also summarized using descriptive statistics. Education was dichotomized into primary/secondary education and higher education because just 1.3% of the respondents received primary education and almost 78% had higher education. The wealth quintile was derived by using principal component analysis with five quintiles (poorest, poorer, middle, richer, and richest). However, the poorest and poorer were recoded as poor and the richer and richest were recoded as rich in our analysis.

Regarding the consumer costs, we estimated two outcome variables. First, the total monthly cost of haemodialysis treatment alone is the sum of drugs, laboratory, transport and feeding costs. The second outcome variable, the total cost of managing ESRD, was derived by summing the monthly cost of haemodialysis and the monthly cost of managing all comorbidities. We assumed that the difference between insured and uninsured patients would be in the level of out-of-pocket spending since insurance covered some drugs and laboratory costs for the insured. We used cross-tabulation to compute the mean costs disaggregated by the respondents’ sociodemographic, economic, and health-related characteristics. The study tested the mean differences with the Mann Whitney U and Kruskal-Wallis tests. Non-parametric tests are appropriate when the costs are skewed, as the Shapiro test showed. Further, we modelled variables (x) that were significant in the bivariate analyses to determine the predictors of the costs using a Generalized Linear Model (gamma with log link). The log-linked function can be viewed as $E\left({y}_{i}{/x}_{i}\right)= \text{e}\text{x}\text{p}\left({\beta }_{i}{x}_{i}\right)$ where E(y) is the expected value of out-of-pocket payment, ${x}_{i}$denotes the set of covariates and β_i_, the estimated coefficients of the explanatory variables. We used gamma with log link because our cost data violated the assumptions of general linear model fitted using ordinary least squares including homoskedasticity, normality, and independence [36]. The level of significance was ρ < 0.05.

### Ethical consideration

The study obtained ethical approval from the Federal Capital Territory Health Research Ethics Committee (FHREC/2019/01/02/10-01-19) and administrative permits from all the participating hospitals. We also obtained informed written consent from respondents before administering data tools. Equally, we anonymized all data before analysis.

## Results

### Sociodemographic and other characteristics of the respondents

Table 1 shows the sociodemographic and health-related characteristics of respondents. Most respondents were male, over 50 years old, married, and residents of Abuja municipality and its satellite towns. Most respondents had higher education, were employed and were in the rich quintiles. About 77% of respondents lacked health insurance coverage. Most respondents had at least four sessions of haemodialysis per month. The commonest facility used for haemodialysis was private (43%). Approximately 92% of respondents had comorbidities. Catheterization was the most common route for haemodialysis treatment.

**Table 1:** Socio-demographic characteristics of respondents (N = 230)

Characteristics		Frequency (n)	Percent (%)
Gender	Male	150	65.2
	Female	79	34.3
	Missing	1	0.4
Age	≥ 60	121	52.6
	40–59	69	30.0
	< 40	40	17.4
Marital status	Single	51	22.2
	Married	145	63.0
	Widowed	21	9.1
	Divorced/Separated	8	3.5
Missing	Missing	5	2.2
Residence	Others	24	10.4
	Satellite	90	39.1
	Nearby towns	45	19.6
	Municipality	69	30.0
	Missing	2	0.9
Education	SSCE and below	51	22.2
	Higher education	179	77.8
Employment	No	40	17.4
	Yes	190	82.6
Income	< 100,000	123	53.5
	≥ 100,000	107	46.5
Wealth	Poor	11	4.8
	Middle	10	4.3
	Rich	209	90.9
Health insurance	No	178	77.4
	Yes	52	22.6
Type of Facility	Private	99	43.0
	PPP	38	16.5
	Public	86	37.4
	Missing	7	3.0
Comorbidity	No	19	8.3
	Yes	211	91.7
TVA	Catheter	180	78.3
	AVF	27	11.7
	Central line access	11	4.8
	Missing	12	5.2
Number of dialysis session	0–3	25	10.9
	4–6	58	25.2
	> 6	141	61.3
	Missing	6	2.6

### Patient costs

Table 2 shows the mean annual spending on haemodialysis treatment and ESRD (haemodialysis and comorbidities). The estimated annual total cost of haemodialysis treatment is $4,447.32. A session costs about $28.51, given an average of thirteen (13) sessions per month. The mean annual cost of ESRD, including morbidities, is $7,415.55. Haemodialysis procedure constitutes about 60% of the estimated total ESRD management cost (including comorbidities). Drugs, laboratory investigations, transport and feeding comprise about 45.8%, 30.5%, 13.3% and 10.4% of the total direct patient cost of haemodialysis treatment. Comorbidities account for about 40% of total monthly spending on ESRD.

**Table 2:** Mean annual patient cost of haemodialysis (HD) and ESRD management (USD)

Cost item	Mean	Std. Deviation	Proportion of Annual HD cost (%)	Proportion of Annual ESRD cost (%)
Drugs	2,037.44	2,523.71	45.8	27.5
Laboratory	1,356.50	1,714.54	30.5	18.3
Transport	589.63	756.29	13.3	8.0
Feeding	463.74	487.16	10.4	6.3
Diabetes	152.08	792.33		2.1
Hypertension	654.94	2,925.39		8.8
Cardiovascular disease	43.79	345.25		0.6
Anaemia	709.52	8,411.54		9.6
Osteoporosis	1.63	19.08		0.0
Sexual dysfunction	2.77	42.05		0.0
Depression	25.41	287.51		0.3
Other comorbidities	1,378.08	3,056.45		18.6
Annual patient HD cost ($)	4,447.32	4,193.88		60.0
Annual patient ESRD cost ($)	7,415.55	12,063.37		
Unit cost HD session	28.51	26.88		

### Factors associated with the cost of managing end-stage renal Disease

The mean direct cost of haemodialysis treatment significantly differs with marital status (ρ = 0.003), residence (p < 0.001), education (p = 0.001), wealth (p < 0.001), health insurance (p = 0.001), type of health facility (p < 0.001), presence of comorbidities (p = 0.001), and number of dialysis sessions (p < 0.001) as shown in Table 3. Also, the direct total cost of managing ESRD (haemodialysis and comorbidities) is associated with age (p = 0.022), marital status (p = 0.002), residence (p < 0.001), education (p = 0.003), wealth (p = 0.001), health insurance (p = 0.001), type of health facility ((p < 0.001), presence of comorbidities (p = 0.001), and number of dialysis sessions (p < 0.001) (Table 3).

**Table 3:** Mean differences in estimated annual haemodialysis and ESRD costs

		Annual HD cost ($)			Annual ESRD cost ($)		
Characteristics		Mean	Std. Deviation	P-value	Mean	Std. Deviation	P-value
Gender^+^	Male	4312.73	3572.09	0.612	7208.25	12872.45	0.878
	Female	4641.85	5185.84		7618.88	10377.66	
Age^++^	60+	4992.02	4778.15		8529.83	9576.69	
	40–59	3966.75	3273.24	0.211	5385.63	5620.94	0.022*
	< 40	3628.56	3511.52		7546.42	22495.55	
Marital status^++^	Single	3485.36	3328.86		7135.92	20184.09	
	Married	4974.63	4399.30	0.003	7850.29	8611.53	0.002*
	Widowed	2759.70	2912.43		4336.88	6354.55	
	Divorced/Separated	2423.68	2096.14		3061.36	2103.14	
Residence^++^	Others	7850.09	5912.97		12615.98	8385.63	
	Satellite	4119.43	4185.71	< 0.001	7970.16	17069.15	< 0.001*
	Nearby towns	2895.60	2820.35		4001.79	4888.22	
	Municipality	4775.33	3632.26		7170.93	7154.60	
Education^+^	SSCE and below	2928.46	2907.44	0.001	4679.72	5661.24	0.003*
	Higher education	4880.06	4405.03		8195.03	13246.21	
Employment^+^	No	4303.22	4974.26	0.308	9514.81	22834.79	0.380
	Yes	4477.65	4024.93		6973.60	8221.84	
Income^+^	< 100,000	4465.41	4321.17	0.642	7816.78	14322.40	0.724
	≥ 100,000	4426.51	4062.79		6954.32	8825.70	
Wealth index^++^	Poor	1536.60	2565.42		2472.62	4963.10	
	Middle	3004.92	2891.83	0.001	3977.16	3438.24	0.001*
	Rich	4669.53	4253.06		7840.22	12509.19	
Health insurance^+^	No	4917.61	4447.64	0.001	8315.52	13318.08	0.001*
	Yes	2837.48	2628.42		4334.86	5034.85	
Type of facility^++^	Private	6860.92	4662.98		12229.03	16497.49	
	PPP	3379.94	2810.87	< 0.001	4558.79	4202.70	< 0.001*
	Public	2141.39	2315.19		3007.24	3887.46	
Comorbidity^+^	No	2612.22	3727.96	0.001	3909.04	6552.63	0.001*
	Yes	4612.56	4201.98		7731.30	12401.55	
TVA^++^	Catheter	4480.95	4285.21		7994.59	13376.86	
	AVF	4632.80	3531.15	0.656	5853.43	4603.41	0.774
	Central line access	4131.34	4484.94		4633.75	4410.92	
No of HD sessions^++^	0–3	3421.24	5653.01		4647.27	6553.31	
	4–6	1512.31	1428.17	< 0.001	2158.67	2507.10	< 0.001*
	> 6	5927.96	4023.09		10252.58	14359.22	

+Mann-Whitney U test; ++Kruskal-Wallis *Significant at p-value < 0.05

### Predictors of cost of haemodialysis

As shown in Table 4, residing in other towns (β = 0.55, ρ = 0.001), lack of health insurance (β = 0.24, ρ = 0.038), attending private health facilities (β = 0.46, ρ < 0.001), using public-private partnership hospital (β = 0.61, ρ < 0.001), and greater than six haemodialysis sessions (β = 0.79, ρ < 0.001) significantly influenced the cost of haemodialysis treatment alone.

**Table 4:** Predictors of annual patient cost of haemodialysis

Parameter		B	Std. Error	95% CI		Hypothesis Test	
				Lower	Upper	Wald Chi-Square	Sig.
	(Intercept)	6.81	0.27	6.27	7.34	618.31	< 0.001
Marital status	Single	0.26	0.25	-0.22	0.74	1.09	0.295
	Married	0.46	0.24	-0.01	0.92	3.73	0.053
	Widowed	0.20	0.27	-0.33	0.73	0.54	0.461
	Divorced/Separated	0^a^					
Residence	Others	0.55	0.17	0.22	0.87	10.59	**0.001** ^*^
	Satellite	0.02	0.11	-0.19	0.23	0.05	0.818
	Nearby towns	0.05	0.14	-0.22	0.33	0.15	0.698
	Municipality	0^a^					
Education	SSCE and below	-0.19	0.13	-0.44	0.05	2.37	0.124
	Higher education	0^a^					
Wealth index	Poor	-0.44	0.24	-0.91	0.03	3.42	0.065
	Middle	-0.25	0.23	-0.70	0.19	1.24	0.266
	Rich	0^a^					
Health insurance	No	0.24	0.12	0.01	0.47	4.31	**0.038** ^*^
	Yes	0^a^					
Facility type	Private	0.61	0.13	0.36	0.86	23.19	**< 0.001** ^*^
	PPP	0.24	0.14	-0.02	0.51	3.21	0.073
	Public	0^a^					
Comorbidity	No	-0.27	0.17	-0.60	0.06	2.62	0.105
	Yes	0^a^					
Number of HD Sessions	> 6	0.79	0.15	0.50	1.08	28.43	**< 0.001** ^*^
	4–6	0.00	0.17	-0.33	0.33	0.00	0.990
	0–3	0^a^					
	(Scale)	0.383^b^	0.04	0.32	0.46		

a. Reference category. *Significant at p-value < 0.05

b. Maximum likelihood estimate.

### Predictors of total cost of managing ESRD (haemodialysis with comorbidities)

Residing in other towns (β = 0.59, ρ = 0.004), attending private health facility (β = 0.75, ρ < 0.001), and greater than six haemodialysis sessions (β = 0.99, ρ < 0.001) significantly influenced the cost of end-stage renal disease (Table 5).

**Table 5:** Predictors of annual patient cost of end-stage renal disease

Parameter		B	Std. Error	95% CI		Hypothesis Test	
				Lower	Upper	Wald Chi-Square	Sig.
	(Intercept)	7.22	0.40	6.44	8.00	327.93	< 0.001
Age	≥ 60	0.08	0.22	-0.36	0.51	0.12	0.730
	40–59	-0.37	0.21	-0.78	0.04	3.10	0.078
	< 40	0^a^					
Marital status	Single	0.16	0.34	-0.50	0.82	0.22	0.636
	Married	0.23	0.29	-0.35	0.80	0.59	0.442
	Widowed	-0.11	0.34	-0.77	0.55	0.10	0.748
	Divorced/Separated	0^a^					
Residence	Others	0.59	0.20	0.19	0.99	8.50	**0.004** ^*^
	Satellite	0.16	0.13	-0.10	0.42	1.44	0.230
	Nearby towns	0.01	0.17	-0.32	0.35	0.01	0.934
	Municipality	0^a^					
Education	SSCE and below	-0.28	0.15	-0.58	0.02	3.29	0.069
	Higher education	0^a^					
Wealth index	Poor	-0.41	0.29	-0.98	0.17	1.91	0.167
	Middle	-0.44	0.28	-0.98	0.10	2.50	0.114
	Rich	0^a^					
Health insurance	No	0.27	0.14	-0.01	0.55	3.62	0.057
	Yes	0^a^					
Facility type	Private	0.75	0.15	0.46	1.05	24.84	**< 0.001** ^*^
	PPP	0.09	0.17	-0.26	0.43	0.24	0.621
	Public	0^a^					
Comorbidity	No	-0.23	0.20	-0.63	0.17	1.27	0.259
	Yes	0^a^					
Number of HD Sessions	> 6	0.99	0.18	0.63	1.34	29.96	**< 0.001** ^*^
	4–6	0.19	0.21	-0.23	0.60	0.77	0.380
	0–3	0^a^					
	(Scale)	0.554^b^	0.05	0.46	0.66		

a. Reference category. *Significant at p-value < 0.05

b. Maximum likelihood estimate

### Healthcare provider direct cost

Table 6 shows the direct costs to providers of haemodialysis treatment. While the variable costs constitute about 83%, the fixed costs accounted for just 17% of the provider’s direct costs. The total provider variable cost per session of dialysis is $103.19. The annualized cost is $16,097.64. The total provider fixed cost per dialysis session is $20.50, constituting 16.58% of the provider cost of haemodialysis. The annualized cost is $3,198. The total provider cost of haemodialysis is $123.69 per session. The Annualized provider cost of haemodialysis is $19,295.64. Supplies were the single most significant driver of provider costs (49%), followed by personnel costs (34.4%) and dialysis machines (16.5%).

**Table 6:** Estimated unit and total annual healthcare costs of haemodialysis

Perspective	Cost category	Item	Cost (USD)	% Provider cost
Provider unit cost	Variable	Personnel	42.57	34.41
		Supplies	60.38	48.82
		Water quality management	0.03	0.02
		Servicing of water plant	0.2	0.16
		Maintenance of dialysis machine	0.02	0.01
		**Sub-total**	103.2	83.42
	Fixed	Rent/month	0.11	0.09
		Dialysis machine	20.39	16.49
		Other equipment	0	0
		**Sub-total**	20.5	16.58
	Total		123.69	
Patient unit cost		Direct (drugs, laboratory investigation, transportation, and feeding)	28.51	
Total cost per session of dialysis			152.20	
Estimated annual cost of dialysis			23742.96	

### Healthcare provider and patient direct cost of haemodialysis

The direct patient and healthcare provider cost of a session of haemodialysis is estimated in this study to be $152.20. The annualized cost for an individual is $23,742.96, while the annual total cost for the studied population of 230 was estimated to be $5,460,879.97. The patient and provider costs formed 18.73% and 81.27% of the total cost, respectively.

## Discussion

This study aimed to estimate the direct costs of managing haemodialysis from both the provider and patient perspective. We found that the annual costs of haemodialysis ($23,742.96) and managing ESRD (26,711.19) are high compared to Nigeria’s GDP per capita of $2,126 [7]. This discussion also highlights the relationship between patient costs and the number of haemodialysis sessions, type of health facility, residing in nearby towns, and health insurance.

The annual direct cost of haemodialysis found in the current study is in line with the annual costs of dialysis reported in a preceding study in Sri Lanka [6]. While our finding is much higher than the average annual cost in Burkina Faso, Ethiopia, Kenya, Sudan, Lebanon, and India [6, 13, 15, 26], it is less than the average annual costs from previous studies in Nigeria, Namibia, Senegal, Democratic Republic of Congo, Namibia, Senegal, South Africa, Chile, China, Hong Kong, and Malaysia [3, 6, 14, 16–20]. The mixed findings on the costs of haemodialysis across different settings are related to varying costing perspectives, costs associated with patient management protocols, currency volatility, timing of studies, and methodology when assessing costs [6].

Consistent with evidence from previous studies [3, 14, 16], direct costs such as dialysis nursing salaries, dialyzers, dialysis machines, blood bags and salaries of nephrologists account for most of the provider costs in the current study. In countries with lower costs, such as Lebanon, the Ministry of Health reimburses the physician’s fee for haemodialysis [15]. In Nigeria, policymakers could consider several cost-reduction strategies. First, transfer provider costs to third-party administrators by expanding the coverage of ESRD patients under the National and State Health Insurance Schemes. Second, reducing the nurse-to-patient ratio might reduce the personnel cost, as proposed elsewhere [14]. The third option is to leverage the 2014 National Policy on Task-shifting and Task-sharing to retrain, equip, and use lower cadres of nursing staff to manage ESRD patients. Moreover, to reduce the cost of supplies and consumables, the government could reduce the import duty taxes on dialysis supplies, similar to the import duty waiver for medicines and health technologies required to manage COVID-19 [37].

In this study, drugs, laboratory tests, transportation, and feeding, subject to increasing inflation in Nigeria, drive patients’ out-of-pocket spending on haemodialysis. Regular stock-out of medicines due to an inefficient inventory management system is commonly encountered in health facilities in Nigeria [38]. Consequently, patients must buy medical products from private pharmacies, often at higher prices than public health facilities [39]. Efforts are needed to reduce the burden of out-of-pocket spending by ESRD patients receiving haemodialysis treatment, mainly because patients’ out-of-pocket spending is higher than Nigeria’s national per capita income. Given this, ensuring adequate availability of medicines and supplies in hospitals offering dialysis services is crucial. Also, providing public subsidies for dialysis [26] or health insurance coverage [28, 29] would be a meaningful change in Nigeria.

The findings of this study revealed that an increasing number of haemodialysis sessions significantly increased the cost from the patient’s perspective. This finding is unsurprising because cost is a function of health resource utilization [6]. Our finding is consistent with the result from an Ethiopian study in which more monthly visits increased the patient’s haemodialysis [17]. In Burkina Faso, cutting down on check-ups and the quantity of drugs purchased [26] minimized patient costs [26]. In resource constrained settings, decreasing the number of weekly sessions can reduce haemodialysis costs since twice-weekly dialysis has non-inferior survival rates compared with thrice-weekly therapy [22, 23]. Aoun et al. argue that a scenario with twice-weekly dialysis for patients with residual kidney function can help lower the total haemodialysis cost and lighten the burden on society and third-party payers [15].

The current study revealed that health facility ownership significantly predicted the cost of haemodialysis. The odds of higher costs were significantly higher among ESRD patients attending private health facilities than public health facilities. Our finding, however, differs from the finding in Burkina Faso, where attending a publicly owned university hospital for haemodialysis treatment was associated with the higher direct cost of dialysis [26]. The possible explanation for the finding of our study is the lack of public subsidy in private health facilities and relatively lower subsidy in public-private partnership health facilities compared to publicly owned health facilities. In Nigeria, the transaction costs of importing kidney care facilities and training costs increased haemodialysis costs in private health facilities [19]. Evidence from South Africa suggests that personnel costs, haemodialysis supplies, outsourcing fees and pharmaceutical supplies are the main dialysis cost drivers in PPP health facilities [14]. Furthermore, vascular access creation, a procedure generally charged for in the private sector, is directly borne by the patient, compared with free or highly subsidized public hospitals [27].

Consistent with findings that the type of place of residence significantly influenced haemodialysis costs, the current study indicates that residing in towns other than the municipality and nearby towns increased patients’ costs. Similar evidence exists in Burkina Faso, the Republic of Congo and China, where residence environment is significantly associated with the costs of dialysis [18, 24, 26]. Patients in rural areas of Burkina Faso spent 33% more money on haemodialysis than those in urban areas due to transportation costs [26]. The current study’s finding is not unexpected because residing in other towns meant that patients accessing haemodialysis in Abuja had increased transportation and feeding costs. Interventions by the government to improve the accessibility of haemodialysis services and reduce patients’ transportation and feeding costs are warranted.

Our findings highlight the influence of health insurance on the cost of haemodialysis among ESRD patients in Nigeria. Lack of health insurance increased the direct cost of haemodialysis among ESRD patients in our study. This evidence is comparable to results from the Lao People’s Democratic Republic, where national health insurance improved accessibility to health service utilization for household members with chronic kidney disease by reducing direct medical costs [28, 29]. Nonetheless, regardless of the health insurance coverage, chronic kidney disease patients and their households could encounter catastrophic health expenditures due to nonmedical spending [28]. Evidence suggests that health insurance reduces the likelihood of catastrophic health expenditures in Nigeria, especially for people with low incomes and the chronically ill [40]. Therefore, expansion of health insurance coverage for ESRD patients is needed.

This study provides cost estimates that can inform the decision to expand the coverage of haemodialysis in Nigeria’s social health insurance schemes. Our evidence could also help determine the cost-effectiveness, cost-benefit analysis and budget impact analysis of haemodialysis in managing ESRD patients in Nigeria. Even with this, the findings may not be generalizable to Nigeria. The study included just six hospitals in Abuja, Federal Capital Territory, a predominantly urban and wealthy population. Further large-scale studies may be necessary to ensure generalizability. Also, since the study is hospital-based, those who did not seek care in the facilities at the time of data collection might have been missed. Furthermore, since the study relied on patients’ self-reported drug, laboratory, transport and feeding costs, recollection bias might be a potential limitation. Finally, indirect patient costs were beyond the scope of this study and are an area for future investigations.

## Conclusion

The purpose of this study, which is to estimate the direct costs of haemodialysis from both the provider and patient perspectives, was achieved. The annual provider costs of managing ESRD and haemodialysis are high. The drivers of provider costs are variable provider costs, including dialysis machines, haemodialysis supplies and human resources. The patient’s out-of-pocket spending on ESRD and haemodialysis is also high. Higher patient costs of managing ESRD and haemodialysis treatment are associated with an increased number of dialysis sessions and the use of private hospitals. Lower costs were more likely among patients residing in nearby towns. Lack of health insurance was also related to higher costs of haemodialysis treatment. This evidence can inform the planning of dialysis services and setting provider payment rates for the National Health Insurance Authority. The cost analysis can also inform future economic evaluation studies to guide resource allocation decisions.

## Data Availability

The datasets used and/or analysed during the current study are available from the corresponding author on reasonable request.

## Abbreviations

CKD: Chronic kidney disease
ESRD: End-stage renal disease
FSSHIP: Formal sector social health insurance programme
GDP: Gross national product
LMICs: Low and middle-income countries
NHIA: National Health Insurance Authority
and PPP: Public-private partnership
